# Superficial temporal artery-to-middle cerebral artery bypass surgery for middle cerebral artery stenosis in a patient with cerebral autosomal-dominant arteriopathy with subcortical infarcts and leukoencephalopathy

**DOI:** 10.1186/s40064-015-1407-7

**Published:** 2015-10-14

**Authors:** Daisuke Muta, Takayuki Kawano, Naoki Shinojima, Junichi Kuratsu

**Affiliations:** Department of Neurosurgery, Kumamoto University Graduate School of Medical Sciences, 1-1-1 Honjyo, Chuoku, Kumamoto, 8608556 Japan

**Keywords:** CADASIL, STA-MCA anastomosis, MCA stenosis, Microbleeds

## Abstract

Cerebral autosomal-dominant arteriopathy with subcortical infarcts and leukoencephalopathy is a rare hereditary small vessel disease. Ischemic events are the main clinical manifestation of this condition. Here, we present a case in which superficial temporal artery-to-middle cerebral artery anastomosis was performed in a patient with cerebral autosomal-dominant arteriopathy with subcortical infarcts and leukoencephalopathy who developed cerebral infarctions caused by severe middle cerebral artery stenosis. Cerebral blood flow and cerebrovascular reactivity were effectively improved using double anastomoses. To our knowledge, surgical revascularization for patients with this condition has not yet been described in the literature. Superficial temporal artery-to-middle cerebral artery anastomosis is effective for patients with cerebral autosomal-dominant arteriopathy with subcortical infarcts and leukoencephalopathy who show marked regional cerebral hypoperfusion.

## Background

Cerebral autosomal dominant arteriopathy with subcortical infarcts and leukoencephalopathy (CADASIL) is a hereditary, monogenic small-vessel disease caused by mutations in the *Notch3* gene on chromosome 19 (Chabriat et al. 2009; Joutel et al. 1996). Cerebral hemodynamics, including cerebral blood flow (CBF) and cerebrovascular reactivity (CVR), are impaired in this small-vessel disease (Chabriat et al. 2000; Huang et al. 2010; van den Boom et al. 2003). At present, there is no specific treatment for CADASIL. Although CADASIL complicated by atherosclerotic stenosis of the major cerebral arteries or carotid arteries has been reported, the potential clinical implications of such lesions seem limited (Choi et al. 2005; Mawet et al. 2011). The present report describes the case of a CADASIL patient who experienced repeated cerebral infarction caused by severe stenosis of the middle cerebral artery (MCA) and considerably diminished cerebral perfusion within an extensive area of the MCA territory.

## Case description

### History

A 54-year-old left-handed man was referred to our hospital with slight motor aphasia and left finger numbness. His cognitive function and intelligence levels were normal. Three years previously, he had been diagnosed with CADASIL by genetic testing for mutation in the *Notch 3* gene. At the time of initial diagnosis, he complained of sensory disturbances on the right side of his body were caused by a lacunar infarction in the left thalamus. There was no significant stenosis of the major cerebral arteries on MR angiography at this time. During the interval between his initial diagnosis and the second infarction, he was not administered any antithrombotic agents.

### Examination

**Fig. 1:**
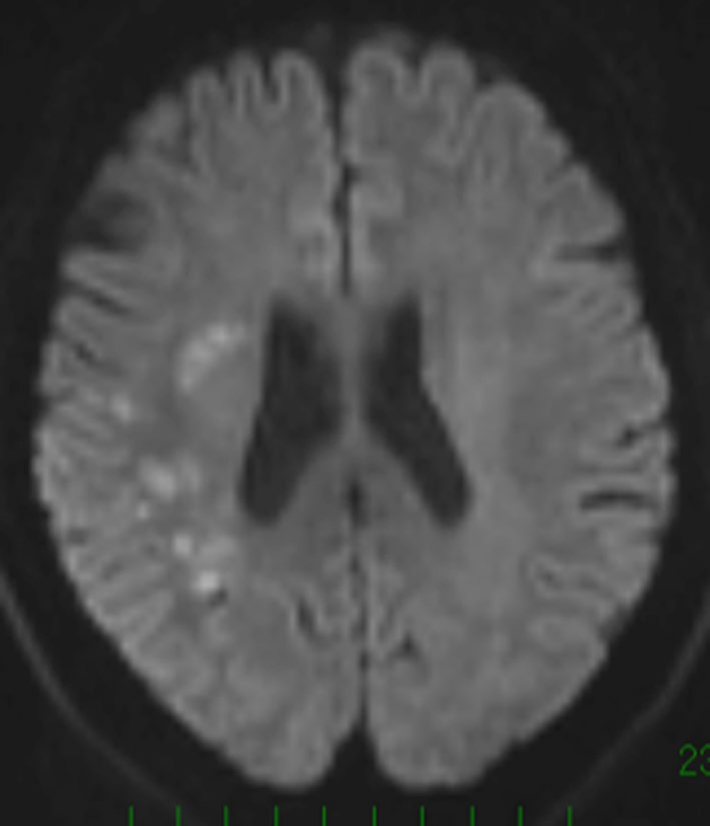
MR imaging (diffusion weighted imaging sequences) revealed multifocal hyperintense signal in a distribution pattern compatible with watershed infarcts in the right posterior MCA territory

**Fig. 2:**
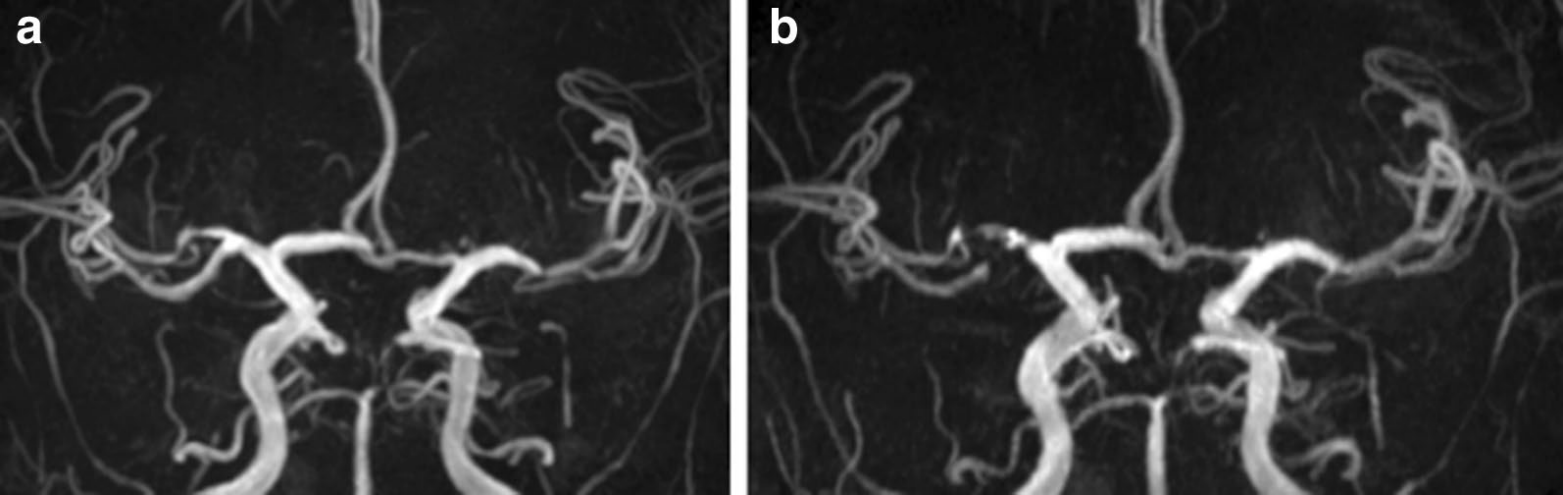
MR angiography showed progression of atherosclerotic stenosis at the right MCA, 6 months before the onset of stroke (**a**) and preoperatively (**b**)

**Fig. 3:**
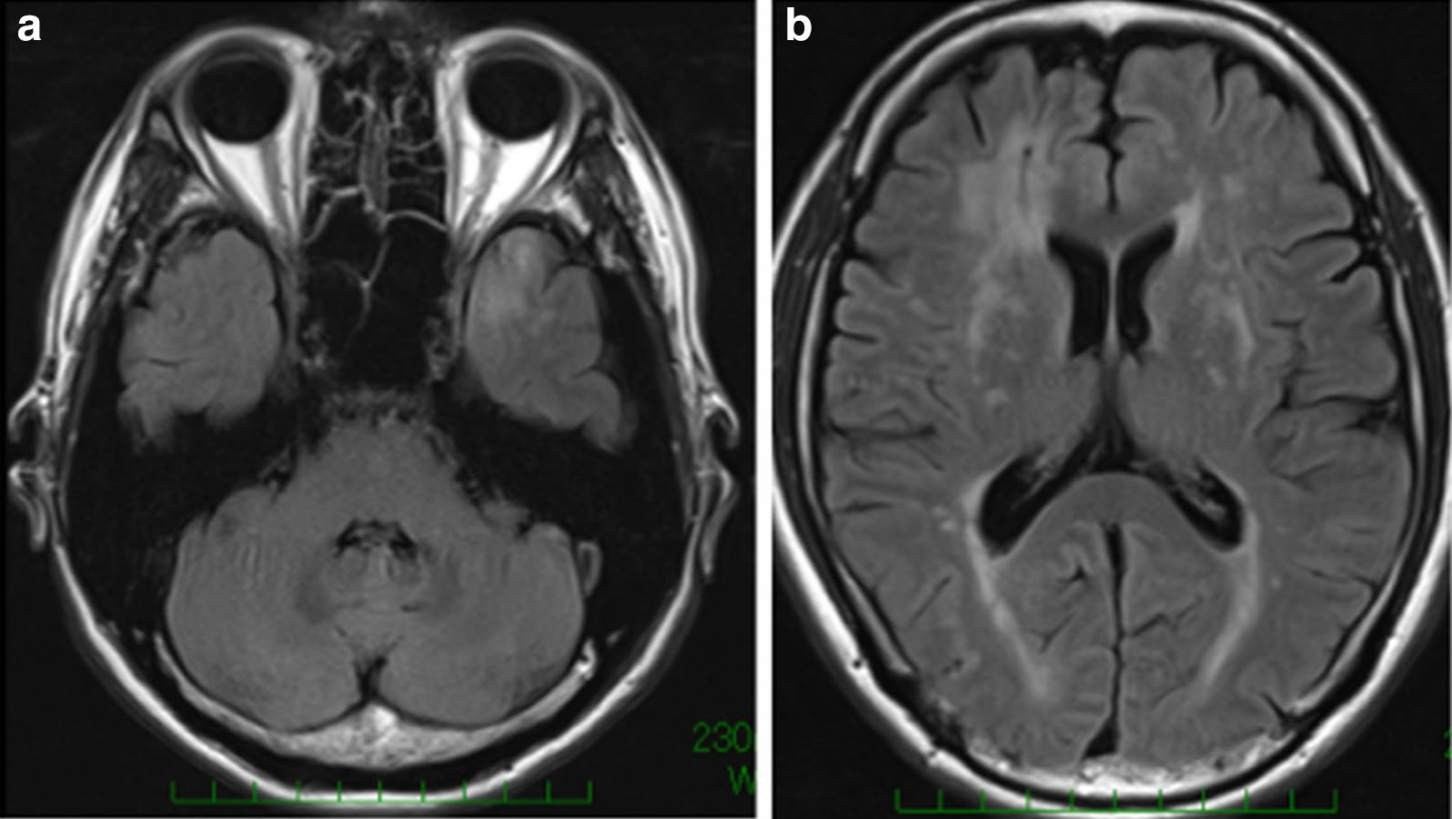
MR imaging (fluid-attenuated inversion recovery) demonstrated typical white matter increased T2-signal intensity abnormalities in the anterior temporal lobe (**a**) and external capsules (**b**)

**Fig. 4:**
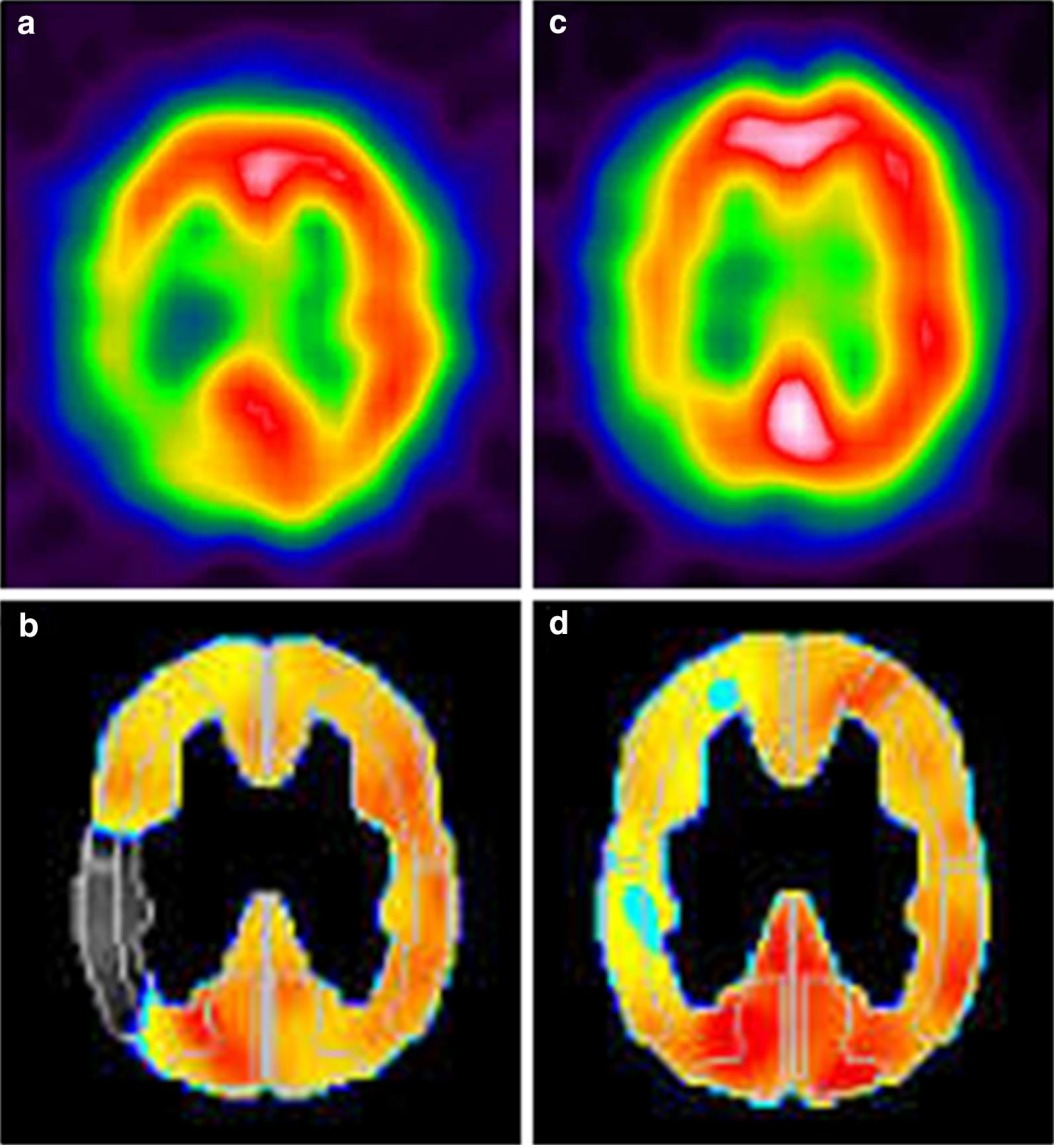
Preoperative SPECT revealed the localized hypoperfusion in the right posterior MCA territory (**a**), and decreased CVR was detected by ACZ challenge (**b**). Improvement of the CBF (**c**) and CVR (**d**) was identified on postoperative SPECT

On clinical examinations, the patient was normotensive. A neurological examination revealed dysphasia, dyslexia, and numbness of the left fingers. Multifocal acute subcortical infarctions were found in the watershed area between the right MCA and the posterior cerebral artery on diffusion-weighted imaging sequences (Fig. 1). MR angiography confirmed severe stenosis of the right MCA that had not been present 6 months prior (Fig. 2a, b). MR imaging (MRI) demonstrated typical increased T2-signal intensity indicating white matter abnormalities in the left anterior temporal lobe, both anterior frontal lobes, external capsules, and periventricular regions (Fig. 3a, b). Some cerebral microbleeds (CMBs) were also present in both the basal ganglia and the subcortical areas. The patient was diagnosed with atherosclerotic infarction and treated with anti-thrombotic therapy. His symptoms were relieved and he was discharged. However, he presented with transient ischemic attacks (TIAs) characterized by left hand motor weakness several times after discharge despite receiving oral antiplatelet treatment. One month after the infarction, [^123^I] *N*-isopropyl-p-iodoamphetamine single photon emission computed tomography (IMP-SPECT) revealed a significant decrease in regional CBF (Fig. 4a) and CVR (Fig. 4b) under the acetazolamide challenge test occurring predominantly in the territory of the right MCA. Therefore, a superficial temporal artery (STA)-MCA anastomosis was planned to improve blood flow on the right side. Preoperative MRI revealed a newly developed cerebral infarction in the right corona radiata (data not shown).

### Surgery

Under general anesthesia, the parietal and frontal branches of the STA were dissected for use as donor arteries. After performing a fronto-temporal craniotomy and dural incision, we exposed two normal-appearing recipient arteries. Intraoperative control indocyanine green (ICG) videoangiography was performed before anastomosis to confirm the patency of the recipient cortical arteries, and showed slow antegrade filling due to atherosclerotic stenosis of the M1 and M2 segments. First, we selected the angular branch of the M4 segment in the region showing a marked decrease in perfusion as a recipient vessel. Anastomosis between the donor and recipient vessels was performed in the usual manner. After ICG videoangiography revealed inadequate filling in the territory of the MCA, an additional anastomosis was performed at the fronto-temporal area of the MCA. The STAs were successfully anastomosed to the recipient vessels. During and after surgery, hypotension and dehydration were avoided, and systolic blood pressure was kept >120 mmHg. Aspirin was administered at a dose of 100 mg/day post operatively.

### Post-operative course

**Fig. 5:**
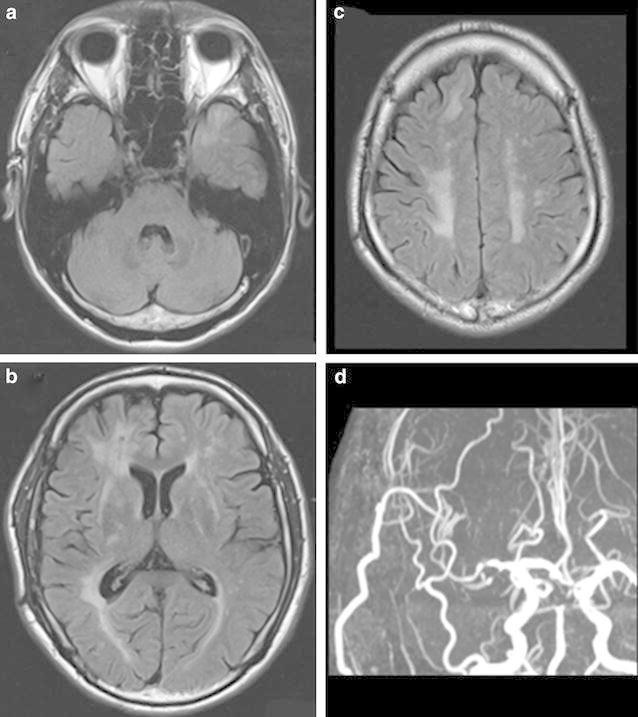
Postoperative MRI shows no remarkable change on abnormal intensity in the anterior temporal lobe (**a**), external capsules, periventricular regions (**b**), and white matter (**c**). MR angiography revealed the patency of the right STA-MCA double anastomoses (**d**)

The post-operative course was uneventful. On post-operative day 1, an IMP-SPECT study showed a 50 % increase in uptake in the right MCA territory but not hyperperfusion, and MRI demonstrated the patency of the double anastomosis (data not shown). IMP-SPECT images obtained 1 month after surgery showed improved regional CBF (Fig. 4c) and CVR (Fig. 4d) in the right hemisphere. No hemorrhagic complications occurred. After surgery, the patient’s preoperative symptoms were relieved, and the TIAs disappeared. The patient was discharged 12 days after surgery. Sixteen months have already passed since surgery, and the patient is doing well. Cerebrovascular events such as TIA, infarction and hemorrhage have not appeared. Follow up MRI 14 months after surgery showed no remarkable area of increased intensity indicating white matter abnormalities in the left anterior temporal lobe, both anterior frontal lobes, external capsules, or periventricular regions on FLAIR images (Fig. 5a–c). MR angiography was used to confirm the patency of the bypass (Fig. 5d).

## Discussion and evaluation

This is the first case report of a CADASIL patient with MCA stenosis who underwent STA-MCA bypass to increase cerebral perfusion in the localized ischemic area. In CADASIL, reductions in both CBF and CVR occur in white matter showing T2-hyperintensity. It has been suggested that the degeneration of vascular smooth muscle cells causes arteriopathy, which leads to cerebral hypoperfusion and impaired autoregulation (Chabriat et al. 2000; Huang et al. 2010; Singhal and Markus 2005; van den Boom et al. 2003). Interestingly, the white-matter hyperintensity in the temporal lobe was found predominant in the left side in this case. This asymmetry of white-matter hyperintensity is very unusual rare in CADASIL, since it would suggest that these lesions do not originate from ischemia, but edema instead. The lower extent observed in the most hypoperfused temporal lobe further support that these lesions are not related to ischemia but mat actually result from edema with blood brain barrier dysfunction. Subcortical lacunar infarcts are typically seen in CADASIL, while watershed infarcts have been reported only sporadically, and the cause is considered to be microcirculatory hypoperfusion. (Gordhan and Hudson 2013) In our case, MCA stenosis was responsible for the watershed infarctions. Furthermore, Chabriat et al. observed that the severity of white matter hypoperfusion measured by MRI might be related to the clinical severity of CADASIL. (Chabriat et al. 2000) Therefore, it was expected that the augmentation of hypoperfusion by major artery stenosis would aggravate the symptom. We consider that the improved perfusion caused by surgical revascularization resulted in palliation of the patient’s progressive symptoms. In the present case, quantitative IMP-SPECT revealed respective improvements in CBF and CVR post operatively. These results indicate that it is possible to use bypass surgery to improve both CBF and CVR in CADASIL patients.

Although the use of antiplatelet drugs for secondary prevention of cerebral ischemic phenomena is common in CADASIL patients. In the present case, the antiplatelet treatment was discontinued since we believe that this was important to reduce the risk of hemorrhagic complications. Recent studies have warned of the increased risk of intracerebral hemorrhage (ICH) with antithrombotic therapy in CADASIL. (Choi et al. 2006; Lian et al. 2013; Oh et al. 2008) CMBs are frequently detected on MRI in CADASIL patients (Lesnik Oberstein et al. 2001). Previous reports have shown that CMBs are associated with an increased risk of ICH (Bokura et al. 2011; Lian et al. 2013; Ragoschke-Schumm et al. 2005; Rinnoci et al. 2013). Although CMBs were also observed in the present case, hemorrhagic complications did not occur after bypass surgery. Surgical treatment allows for the discontinuation of antiplatelet treatment, and we believe that this reduces the risks of hemorrhagic complications.

In addition, strict control of blood pressure is required in the peri- and post-operative periods. Choi et al. recently found that CADASIL patients with hypertension had a significantly higher prevalence of stroke, and experienced significantly greater numbers of CMBs than those without hypertension (Choi et al. 2006). In contrast, an excessive decrease in blood pressure should be avoided because it can lead to cerebral infarction caused by hypoperfusion. In particular, blood pressure drops during surgery due to anesthesia should be treated with caution. Chui et al. described the anesthetic considerations for bypass surgery. The general recommendation is to maintain “normotension” or to keep the blood pressure within 10–20 % of the preoperative established baseline blood pressure for all patients. (Chui et al. 2015)

Although bleeding risks caused by postoperative hyperperfusion were a concern, bypass surgery resulted in no post-operative problems and improved the patient’s symptoms. Because surface structure abnormalities of the recipient arteries were not seen during surgery, the vascular walls of the recipient arteries did not affect the anastomotic maneuvers. These findings are consistent with a previous study, which reported that cortical artery vascular wall abnormalities were rare in CADASIL.

## Conclusions

We conclude that although strict management of blood pressure during and after surgery is necessary, STA-MCA bypass is an effective and safe strategy for the management of CADASIL patients in whom stenosis of the large cerebral arteries causes a risk of cerebral infarction because of hypoperfusion with impaired CVR.

## Abbreviations

CADASIL: cerebral autosomal dominant arteriopathy with subcortical infarcts and leukoencephalopathy
CBF: cerebral hemodynamics, including cerebral blood flow
CVR: cerebrovascular reactivity
MCA: middle cerebral artery
CMBs: cerebral microbleeds
IMP-SPECT: [^123^I] *N*-isopropyl-p-iodoamphetamine single photon emission computed tomography
STA: superficial temporal artery
ICG: indocyanine green
ICH: intracerebral hemorrhage
